# Large-scale evaluation of interventions designed to reduce childhood Drownings in rural Bangladesh: a before and after cohort study

**DOI:** 10.1186/s40621-020-00245-2

**Published:** 2020-05-11

**Authors:** Olakunle Alonge, David Bishai, Shirin Wadhwaniya, Priyanka Agrawal, Aminur Rahman, Emdad Md. Dewan Hoque, Kamran Ul Baset, Shumona Sharmin Salam, Al-Amin Bhuiyan, Md Irteja Islam, Abu Talab, Qazi Sadeq-ur Rahman, Fazlur Rahman, Shams El-Arifeen, Adnan A. Hyder

**Affiliations:** 1 Johns Hopkins International Injury Research Unit, Department of International Health, Johns Hopkins Bloomberg School of Public Health, Baltimore, USA; 2 Department of Population and Reproductive Health, Johns Hopkins Bloomberg School of Public Health, Baltimore, USA; 3 Center for Injury Prevention Research, Dhaka, Bangladesh; 4 Maternal and Child Health Division, International Center for Diarrhoeal Diseases Research, Dhaka, Bangladesh; 5 Milken Institute School of Public Health, George Washington University, Washington DC, USA

**Keywords:** Drowning, Childhood, Intervention, Daycare, Creche, Effectiveness, Bangladesh, Low- and middle-income countries, Observational study

## Abstract

**Background:**

This paper estimates the impact on childhood drowning rates of community-based introduction of crèches or playpens or both in rural Bangladesh for children aged 0–47 months.

**Methods:**

A baseline census of the whole population of 270,387 households in 51 unions, 451 villages from 7 rural sub-districts in Bangladesh was conducted in 2013. The baseline census determined retrospective, age-specific, and cumulative drowning incidence rates (IR) experienced in the target households in the 12 months prior to the intervention. Beginning in late 2013, creches for drowning prevention were established across the study area. Acceptance into creches was provided and written assent to attend a creche was obtained for all children aged 9–47 months in all participating unions. Playpens were provided to 45,460 of these children, of which 5981 children received only the playpens. All children were followed-up until their 48-month birthday or administrative censoring (fixed timepoint to stop observing the drowning deaths), after a two-year implementation period (2014–2016). Drowning IR were estimated for children and compared to corresponding baseline rates from 2012. Age-specific drowning IR under different “as treated” categories (playpen-only, creche-only, and playpen-plus-creche) were compared to the baseline rates experienced by the categorized households prior to intervention.

**Results:**

A total of 3205 creches (average of 7 creches per village) were established, and 116,054 children aged 9–47 months were exposed to the intervention packages. Aggregated drowning IRs between age 0 and 47 were estimated per 100,000 population per year at 86.73 (95% CI: 69.67–107.97) and 43.03 (95% CI: 35.55–52.10) in the baseline and post implementation period, respectively. Risk ratios were 0.40 (95% CI: 0.28–0.57) overall, and 0.34 (95% CI: 0.13–0.90), 0.09 (95% CI: 0.02–0.36), and 0.04 (95% CI: 0.002–0.60) in children under the creche-only, aged, 1, 2, and 3 years old respectively. Inexplicably, drowning rates were statistically significantly higher post-intervention in children 0-11 months. There was no mortality reduction with playpen use (alone or in combination), and this group may actually have had a higher risk of drowning.

**Conclusions:**

Creches are effective for preventing childhood drowning in rural Bangladesh for children above age 1-year, and should be considered for further scale-up.

## Introduction

Deaths from communicable diseases have declined over the last decade globally, but the proportion of deaths resulting from injuries has steadily increased over the same period (Liu et al. 2012). Drowning is the second leading cause of injury-related deaths among children aged 1–19 years worldwide (World Health Organization 2016; World Health Organization 2014). About 359,000 people die from drowning annually, 97% of these deaths occur in low- and middle-income countries (LMICs), and about 20% among under-five children (World Health Organization 2016; World Health Organization 2014). Bangladesh has one of the highest under-five drowning rates in the world (World Health Organization 2016; World Health Organization 2014). The country achieved the Millennium Development Goal of a two-third reduction in under-five mortality by 2015, largely efforts to reduce infant deaths (Chowdhury et al. 2013). However, children who survive through infancy face a high risk of death from drowning later on in life (National Institute of Population Research and Training (NIPORT) 2016). About two children die every hour from drowning in Bangladesh (Rahman et al. 2005); and for children aged 12–47 months, drowning is the leading cause of mortality accounting for 42% of all deaths in this age group (National Institute of Population Research and Training (NIPORT) 2016). The current under-five mortality profile in Bangladesh reflects the ongoing epidemiological transition in LMICs, and the need to rethink interventions for child survival (Alonge et al. 2017a).

Specific geographical and sociocultural factors make children in Bangladesh particularly prone to drowning (Ahmed et al. 1999; Gain et al. 2002). Ninety percent of Bangladesh is a floodplain, and the country has the world’s largest delta and highest density of rivers per area (Gain et al. 2002). To prevent flooding, most homes in Bangladesh are built on elevated land mass created from excavating around home sites leaving behind uncovered pits that later become ditches and ponds adjoining homes. The majority of drowning among under-five children in Bangladesh takes place in these ditches and ponds less than 20 m from homes during the morning hours (9 am – 1 pm) when caregivers are often busy with household chores (Ahmed et al. 1999; Hyder et al. 2008). Other factors consistently associated with childhood drowning in Bangladesh include age, rural residency and the monsoon rainy season (April–September) (Ahmed et al. 1999; Hyder et al. 2008). Lack of adequate supervision underlies most direct causes of childhood drowning and is associated with 70% of drowning deaths among under-five children in Bangladesh (Hyder et al. 2008; Petrass et al. 2011; Ya et al. 2007). Hence, active supervision of children has been suggested as a possible solution to the high rates of drowning in rural Bangladesh (Hyder et al. 2008), although this mechanism may not address all of the known risk factors for childhood drowning.

Based on a comprehensive review of the state of knowledge in drowning prevention (World Health Organization 2014; Peden et al. 2008), and empirical evidence from pilot studies (Hyder et al. 2008), the following set of interventions have been proposed for drowning prevention among under-five children: 1) wearing of personal protection devices, 2) fencing of water bodies, 3) use of door-barriers or playpens, and 4) supervision of children in crèches. Most of these interventions however lack evidence of effectiveness in a LMIC setting (World Health Organization 2014; Hyder et al. 2008; Peden et al. 2008).

A previous study from Bangladesh demonstrated that under-five children who participated in a packaged program (including enrollment in crèches, home visits for safety education, community education on injury prevention, establishment of village injury prevention committees, social autopsy on injury deaths and use of playpens) were 82% less likely to drown compared with those who did not participate (Rahman et al. 2012). However, it was difficult to establish which component of the packaged program led to the observed effect given that there were several interventions involved.

The primary goal of this current study, called **S**aving **o**f **Li**ves from childhood **D**rowning (SoLiD) program, is to measure the large-scale impact of a streamlined package of drowning interventions on overall and age-specific drowning mortality rates. The package comprises largely three components: a creche-only, a playpen-only, and a creche plus playpen component; each combined with the establishment of injury prevention committees to provide community education on injury prevention. A secondary goal of the study is to determine which sub-component of the intervention package may have had the most impact. It is hoped that this study will fill the knowledge gap on evidence for large-scale effectiveness and implementation of drowning prevention interventions among under-five children in LMICs.

## Methods

### Study area

The study was implemented in seven purposively selected rural sub-districts of Bangladesh – Matlab North, Matlab South, Daudkandi, Chandpur Sadar, Raiganj, Sherpur Sadar, and Manohardi (Hyder et al. 2014a; Hyder et al. 2014b). The seven sub-districts were selected because they represented geographically diverse rural populations with potentially high childhood drowning rates, and included sites with past history of demographic surveillance to facilitate data collection for the study (Alonge et al. 2017b). The study area included 51 unions out of 83 total unions in those seven sub-districts. A union is the smallest administrative and local government unit in Bangladesh. The 51 unions included 451 villages and had an estimated population of 1.2 million people in 2011 (International Centre for Diarrheal Disease Research, Bangladesh (icddr, b) 2007).

### Study procedure and implementation

During the study start-up period (October 2012 – May 2013), stakeholders’ advocacy meetings were led by trained project staff at the sub-district, union and village levels (including, 7 sub-district meetings, 51 union meetings and 451 village meetings). The stakeholders at the meetings included political leaders, representatives appointed to sub-district and union councils, and community leaders. The meetings were conducted to sensitize the communities to the high burden of childhood drowning in Bangladesh, and to raise awareness on the need for action.

Over the next period (June 2013 – November 2013), a baseline census was conducted to cover the entire study area, including 51 unions and all 1.2 million people. The baseline census collected information on social and demographic characteristics, care-seeking behavior, health outcomes, including retrospective mortality by cause for children who died between ages 0 and 47 months in the 12 months prior to the census (Alonge et al. 2017b). Trained data collectors surveyed heads of households or any adult 18 years and older using a questionnaire covering seven modules, including an injury mechanism module that described drowning death. Drowning death was defined as injury death from respiratory impairment resulting from submersion of the face in water (van Beeck et al. 2005). Further details on the methodology and results from the baseline census are described elsewhere (Alonge et al. 2017b).

Following the baseline census, an active demographic and injury surveillance system was maintained across the entire study area over the remainder of the study period, between October 2013 and February 2016, which overlapped the intervention implementation period. The surveillance system captured all births, household migrations, and deaths, including drowning deaths for all children in the study area. All households were visited every 4 months, and six rounds of surveillance were completed over the course of the study using both paper-based and tablet-based data collection and entry systems. To ensure data quality, 10% of all interviews were observed by data supervisors (and 2% of these were re-interviewed). Ten percent of completed paper forms were randomly selected and reviewed for any inconsistencies, and validation rules were set to prevent inconsistencies and errors during the data entry for the tablet-based system. Monthly data quality assurance meetings were conducted between data collectors and supervisors to further review the paper-based data. The central database was also reviewed frequently for any inconsistencies, and these inconsistencies were corrected after discussion with the field team in real-time.

Prior to the roll out of the intervention package, additional rounds of stakeholders meetings were conducted by project staff in each of the 7 sub-districts, 51 unions and 451 villages starting from October 2013. These meetings were conducted to sensitize the stakeholders to the package of interventions, and to establish injury prevention committees at the union and village levels. The union injury prevention committees (UIPCs) and village injury prevention committees (VIPCs) were established to provide ongoing community education on injury prevention, and to facilitate childhood drowning and injury prevention activities in their communities. Each UIPC comprised of elected representatives from that union, including a chairman and 10 other council members. Each VIPC included 5–7 members, comprising elected representatives, community leaders and parents of young children. All UIPC and VIPC members received a half-day orientation and training on injury and drowning prevention provided by project staff. The UIPCs and VIPCs were assigned to meet once every month to discuss and plan injury prevention activities, and their activities continued throughout the study period. Annual meetings were also held at the sub-district levels to appraise political leaders on the progress of the project and to discuss implementation issues that required political support.

Based on the recommendations of each VIPC, private homes of selected female community members were identified as creches for childhood drowning prevention for children in the study area. These crèches were tuition-free daycare centers that operated between 9 am and 1 pm daily, 6 days a week, and were required to meet specific criteria. A creche should consist of a room with secured doors and windows, clean and carpeted floors, adequate light and ventilation, and be equipped with age appropriate toys and educational supplies. Each creche was supervised by two trained workers (a crèche mother and her assistant). The creche workers were trained to keep all children within reach and under direct visual contact at all times. They were also trained on early childhood development methods, injury prevention, record-keeping (including keeping track of the number of children), and how to maintain adequate health and safety standards. Each creche was staffed to accommodate 24–30 children (Hyder et al. 2014a; Hyder et al. 2014b). The creche mother and her assistant were paid 25-27USD and 7-8USD honorarium per month for their activities, respectively. Daily activities in the crèche included early childhood learning programs, social activities such as singing and dancing, and instructions on health hygiene (Hyder et al. 2014a; Hyder et al. 2014b). The hypothesized mechanism of action by which a crèche prevented childhood drowning was to keep at-risk children under adult supervision, and away from water hazards during the peak period (9 am – 1 pm) when drowning was most likely to occur (Rahman et al. 2012).

In addition to the creches, playpens were distributed to some caregivers in the study area. The playpens were rigid four-sided enclosures made of wooden or plastic slats and a firm base (Hyder et al. 2014a; Hyder et al. 2014b). Both the wooden and plastic playpens were designed to conform with standards and guidelines set by the United States Consumer Protection Safety Commission for Playpens/Play-yards (United States CPSC n.d.). Wooden playpens were heavier with more slats, a thicker base and fixed joints. The plastic playpen did not have fixed joints and could be readily assembled and disassembled by caregivers. The caregivers were encouraged to use the playpen as a supervisory aid to keep children from water hazards. They were instructed to keep only one child in a playpen at a particular time. Other instructions provided to the caregivers on the use of the playpen are described in the supplementary file (Appendix).

To facilitate rapid and large-scale roll-out of the intervention package, the study area was divided into two halves (Area 1 and 2). Area 1 comprised 3 sub-districts: Raiganj, Manohardi and Sherpur sub-districts. Area 2 comprised 4 sub-districts: Matlab North, Matlab South, Daudkandi and Chandpur sub-districts. No sub-district in Area 1 borders a river, but all of the districts in Area 2 border either the Padma or Meghna river. Area 1 was supported by one non-governmental organization (NGO) and Area 2 had a different NGO. Each NGO was responsible for data collection and delivering the intervention package in their respective area. Both NGOs had experience working in the area that they were assigned, and had a long familiarity with the local communities.

All children aged 9–47 months (the age bracket with the highest risk of drowning deaths in Bangladesh) (Hyder et al. 2014a; Hyder et al. 2014b) within both areas were eligible to participate in the creches and/or use of playpens. Eligible children were identified during the baseline census and surveillance rounds, and individual written informed assent to attend the creches and/or use the playpens was obtained for all children 9–47 months in the study during the census and surveillance. (A separate informed assent was obtained for research participation in the baseline census and surveillance prior to conducting the census.) VIPC members and crèche mothers liaised with head of households in their communities to ensure that all eligible children in their villages had parents who knew that their children had been accepted for enrollment in the creches. In both areas, playpens were also distributed to eligible children in addition to the creche intervention. The playpens were distributed on a first-come first-served basis from a central village distribution point or from the creche. In Area 2, 5981 children aged 9–24 months had parents who selected to participate in only the playpen intervention. We achieved an intention to treat 95% of children by successfully informing the parents of every eligible child that they had been accepted into the local crèche. The VIPC members and project staff conducted periodical visits to the crèches, and to families with playpens to monitor the performance of the interventions over time. Project staff and VIPC members were also monitoring the creches for any outbreak of infectious diseases.

All children in the study areas were followed up until they reached 48 months of age or were administratively censored (that is, a fixed timepoint to stop observing the drowning deaths) on October 6th, 2015 and November 3rd, 2015 in Area 1 and Area 2, respectively. The censor dates marked the end of 2 years of implementing the interventions in both areas. Based on the uptake of interventions, all children aged 9–47 months in the study area could be classified under 3 different treatment categories: creche-only, playpen-only, and creche plus playpen categories; each combined with the establishment of injury prevention committees to provide community education on injury prevention at various levels.

### Study design

The primary goal was to estimate the change in drowning deaths. A pre-post design was used to compare fatal drowning outcomes for children overall and by age group and by study area between pre-intervention and post-intervention periods. We specifically compared cumulative incidence of drowning at baseline assessed over one-year recall from 2012 to 2013 with annualized cumulative incidence of drowning during surveillance from 2013 to 2015 after implementing the interventions.

A secondary goal was to attempt to determine which components of the intervention were the most effective—playpen-only, creche-only or creche plus playpen. Achieving this secondary goal was much more challenging because playpen acquisition (and use) and creche attendance were subject to self-selected uptake. Ultimately, parents and caregivers decided how much to use playpens and creches. Because it was a pragmatic trial, the study did not establish a group of post-intervention controls who were denied entry to creches and/or denied access to playpens. Given the limitations of drawing inferences in the presence of selective intervention uptake, we still pursued our secondary goal by comparing cumulative incidence of drowning of children under the different treatment categories with cumulative incidence of drowning of their older siblings (or younger versions of the treated children) during the 12 months prior to the baseline census. We classified households in the baseline data into categories based on the treatment (creche-only, playpen-only, or creche plus playpen) that they ultimately entered by the end of the study. We thus calculated baseline drowning rates by treatment category by examing survival patterns of the untreated older siblings and younger versions of the treated children (i.e. eligible children identified at baseline and who would ultimately receive various categories of the treatments by the end of the study).

Ex-ante sample size calculation suggested that at least 24,000 children would be needed under each treatment category to have 80% power of detecting a 50% decline in drowning specific incidence rate compared to baseline rates.

### Data analysis

Although treatment with playpens and creches was not provided to children under age 9 months, we calculated drowning incidence from birth until 48 months in order to detect spillover effects of the community education on children for whom that was the main treatment. Drowning rates are known to be low during the first year of life, thus we fully expected that precision and statistical power would be lower when comparing drowning incidence across groups under the age of one.

Data from the baseline census were used to estimate cumulative incidence of drowning per 100,000 population per year for all children less than 48 months in the study area, and these were categorized by sex, age group, and implementation area (Area 1 or 2) as well as the treatment eventually received by that household after the creche and playpen interventions started. Similarly, cumulative incidence of drowning per 100,000 population per year was estimated for all children less than 48 months in the study area during 2 years of exposure to the interventions using the surveillance data. The cumulative incidence of drowning from the surveillance data during intervention were further categorized by sex, age group, implementation area (Area 1 or 2) and by the treatment that was received. Comparing rates across these categories of demography and treatment condition offers a “within-household-type” comparison of drowning rates that helps to attenuate self-selection bias due to household specific factors driving selection into treatment. If there were factors leading some households to be more (or less) interested in pursuing the use of creches and playpens these same factors would be attached to the observations of drowning risk of the older siblings (or younger versions of the treated children) making up the untreated comparison groups reporting drowning events and exposure to risk in the year prior to baseline. At the time of the baseline data collection, nobody could predict which household would ultimately select into creche-only or playpen-only or both. However, ex-post, that classification could be made retrospectively and this permits the baseline data to help serve as a control group for the respective treatment groups.

Unadjusted cumulative incidence ratios were estimated comparing estimates from the baseline and surveillance data. Using pooled baseline and surveillance data, Poisson regression model for panel data was used to estimate adjusted cumulative incidence ratios for children aged 9–47 months under each treatment category. The Poisson model used generalized estimating equations (GEE) with population-averaged estimates. The Poisson model adjusted for sex, age group, and implementation area (Area 1 or 2). The standard errors were adjusted for heteroscedasticity using options for robust standard errors with exchangeable autocorrelation function. All statistical analysis was implemented in Stata 14 I/C (StataCorp 2015).

The pre-specified protocol for this study included a cross-over design in which children in Area 1 would begin with the playpens and those in Area 2 would begin with the creches in year 1, and adding a second intervention (creches for Area 1 and playpens for Area 2) in year 2 (Hyder et al. 2014a; Hyder et al. 2014b). However, due to significant delays in production of playpens in year 1, children in both Area 1 and 2 were started on the creches in year 1, except for the 5981 children that received the playpen-only. Other deviations to the pre-specified protocol included the use of a one-year recall period for estimating drowning incidence at baseline (as opposed to a five-year recall period) (Hyder et al. 2014a; Hyder et al. 2014b). The change in the baseline recall period was made because the five-year period was found to be unreliable and subject to a more significant recall bias for estimating mortality compared to a one-year period. Since the baseline recall period was limited to 1 year (instead of 5 years), we could not observe the actual time to event for the baseline assessment. Therefore, we used Poisson regression to model the pre-post incidence rate (and count) of drowning mortality rather than to model the time to events (using the Cox Proportional model as pre-specified) (Hyder et al. 2014a).

#### Ethical approval

Ethical approval for this study was obtained from the Institutional Review Boards of the Johns Hopkins Bloomberg School of Public Health, the Center for Injury Prevention Research, Bangladesh and International Center for Diarrheal Disease Research, Bangladesh.

## Results

Fifty-one UIPCs were established, 561 UIPCs members were trained on childhood injury and drowning prevention activities, 800 VIPCs (1–2 VIPCs per village) were established, and 5600 VIPCs members were trained. A total of 3205 creches (average of 7 creches per village) were established over the study period, and 55,790 playpens were procured and distributed; 38,971 of these playpens were wooden playpens (and were all distributed in Area 1), while the remainder were plastic playpens (distributed in Area 2). A total of 1600 creches were established in Area 1, and 1605 in Area 2.

The baseline census conducted in 2013 included 92,940 children aged 0–47 months, 91% of these were children aged 12–47 months and 51% were male (Table 1). Eighty fatal drowning events were recorded during the one-year period preceding the baseline census for all children age 0–47 months, 78 (98%) of these events were recorded among children aged 12–47 months. The post 2013 surveillance study included 122,032 children aged 0–47 months, and recorded 105 fatal drowning events over a 2 year period. Eighty-seven percent of these children were aged 12–47 months, and 86 (82%) of the drowning events were recorded in this age group. The cumulative incidence of drowning was 86.73 (95% CI: 69.67–107.97) and 43.03 (95% CI: 35.55–52.10) per 100,000 population per year for children aged 0–47 months during the baseline and implementation period, respectively. This represents a cumulative incidence ratio of 0.40 (95% CI: 0.28–0.57) comparing baseline to implementation period (Table 1).

**Table 1:** Frequency and cumulative incidence of drowning in children aged 0–47 months

Variables	Baseline Period (2012–2013)			Implementation Period (2014–2016)			Pre-post comparison	
	Population n (%)	Drowning^**a**^ n (%)	Cumulative incidence per 100,000 population per year (95% CI)	Population n (%)	Drowning^**b**^n (%)	Cumulative incidence per 100,000 population per year (95% CI)	Cumulative incidence ratio (95% CI)	***P***-value
**All children, 0 – 47 months**	92,240 (100)	80 (100)	86.73 (69.67, 107.97)	122,032 (100)	105 (100)	43.03 (35.55, 52.10)	0.40 (0.28, 0.57)	0.0000
**Sex**								
Male	46,857 (50.80)	43 (53.75)	91.77 (68.07, 123.72)	61,801 (50.65)	60 (57.14)	48.56 (37.71, 62.53)	0.53 (0.33, 0.84)	0.0065
Female	45,383 (49.20)	37 (46.25)	81.53 (59.08, 112.51)	60,226 (49.35)	45 (42.86)	37.37 (27.90, 50.04)	0.47 (0.28, 0.79)	0.0035
**Age**								
0–11 months	21,972 (23.82)	2 (2.50)	9.10 (2.28, 36.39)	15,222 (12.48)	19 (18.10)	62.41 (39.82, 97.81)	7.21 (1.58, 32.92)	0.0028
12–23 months	23,117 (25.06)	29 (36.25)	125.45 (87.19, 180.47)	30,350 (24.87)	44 (41.90)	72.51 (53.97, 97.41)	0.58 (0.33, 1.00)	0.0050
24–35 months	24,276 (26.32)	26 (32.50)	107.10 (72.93, 157.26)	38,650 (31.67)	26 (24.76)	33.64 (22.91, 49.40)	0.31 (0.16, 0.61)	0.0003
36–47 months	22,875 (24.80)	23 (28.75)	100.55 (66.82, 151.26)	37,810 (30.98)	16 (15.24)	21.17 (12.97, 34.55)	0.21 (0.09, 0.47)	0.0000
**Study area**								
Area 1	41,785 (45.30)	22 (27.50)	52.65 (34.67, 79.95)	51,569 (42.26)	20 (19.05)	19.41 (12.52, 30.07)	0.37 (0.18, 0.78)	0.0064
Area 2	50,455 (54.70)	58 (72.50)	114.95 (88.88, 148.67)	70,463 (57.76)	85 (80.95)	60.32 (48.77, 74.59)	0.53 (0.36, 0.79)	0.0014

^**a**^Drowning information obtained over 1-year recall period

^**b**^Drowning information obtained over an average 2-year implementation period

There were 19 drowning deaths recorded among those aged 0–11 months during the implementation period compared to 2 deaths at baseline. Ten of these 19 deaths occurred among children 9 months or older, and 16 of these deaths occurred in Area 2. Accepting the data at face value, this would correspond to a heightened risk ratio of 7.21 (95% CI: 1.58–32.92) in the 0–11 month age group during the intervention period. For all other age groups, the risk was reduced during the intervention period (Table 1).

During the implementation period, 5981 children were exposed to the playpen-only and did not attend creches. On an intention to treat basis, 70,594 and 39,479 received creche-only and creche plus playpen treatment, respectively (Table 2). Of the 110,073 that received to any type of creche treatment, 64,096 (58%) ever attended a creche. There was no report of any disease outbreak in the creches during the study period, and less than 0.5% of children that ever attended a creche stopped attending due to any type of illness. Of those who ever attended a creche, 63% of children attended the creches regularly (5 or more days per week for 4 h per day (9 am – 1 pm)), 27% attended often (2–4 days per week), and 10% infrequently (less than 2 days per week). Each creche had an average of 20 children. There was poor compliance with the use of the playpens. Follow up surveys and home site visits showed that 84% of those who received only the playpens did not use them during 8–9 out of 10 home site visits on average. Out of those not using their playpen, 75% reported that the children do not like to stay inside playpen.

**Table 2:** Pre-post comparison cumulative incidence of drowning by treatment category

Variables	Baseline Period (2012–2013)			Post Implementation Period (2014–2016)			Pre-post comparison	
	Population (***N*** = 48,561) n (%)	Drowning^**a**^ (***N*** = 78) n (%)	Age-adjusted cumulative incidence per 100,000 population per year (95% CI)	Population (***N*** = 122,032) n (%)	Drowning^**b**^ (***N*** = 105) n (%)	Age-adjusted cumulative incidence per 100,000 population per year (95% CI)	Cumulative incidence ratio^**c**^ (95% CI)	***P***-value
**Treatment category**^**d**^								
Playpen only (aged 9–35 months)	1150 (2.36)	0 (0)	0	5981 (4.90)	22 (20.95)	183.92 (121.15, 279.03)	0	0
Crèche only (aged 9–47 months)	37,769 (77.78)	78 (100)	206.10 (138.70, 318.00)	70,594 (57.85)	66 (62.86)	46.15 (28.86, 74.34)	0.12 (0.05, 0.29)	< 0.0001
Dual treatment (aged 9–47 months)	8592 (17.69)	0 (0)	0	39,479 (32.35)	8 (7.62)	10.13 (5.07, 20.30)	0	0

^**a**^Drowning information obtained over 1-year recall period

^**b**^Drowning information obtained over an average 2-year implementation period

^c^Cumulative incidence ratios was adjusted for sex, age, and study area using Poisson regression model with Generalized Estimating Equations (GEE)

^d^Children aged 0–8 months were not assigned to any intervention under the treatment arm

There were 48,561 older siblings (or younger versions of the treated children) aged 0–47 months that were assigned to a treatment category at baseline. A total of 78 (out of the total of 80 drowning deaths at baseline) occurred among households with children who ultimately received the creche-only treatment by the end of the study. No deaths occurred among those households with children who ultimately received the playpen-only or creche plus playpen category (Table 2).

The adjusted cumulative incidence ratio for the creche-only category was 0.12 (0.05, 0.29) comparing children who received the intervention with their older siblings (or younger versions) (Table 2). This was statistically significant at 5% alpha level suggesting the creche reduced drowning risk by 88%. Table 3 shows the adjusted cumulative incidence ratio for creche-only by age group. The adjusted age-specific ratios were statistically significant and suggested that the creche reduced drowning risk for all under five age groups except for those aged 0–11 months, and the effectiveness of the creche for drowning prevention increased with age (Table 3).

**Table 3:** Pre-post comparison of age-specific cumulative incidence for children under creche

Age group	Creche only			
	Unadjusted		Adjusted^**a**^	
	Cumulative incidence ratio (95% CI)	*P*-value	Cumulative incidence ratio (95% CI)	*P*-value
0–11 months	5.95 (0.77, 45.80)	0.087	7.00 (0.85, 57.83)	0.071
12–23 months	0.50 (0.30, 0.84)	0.009	0.34 (0.13, 0.90)	0.030
24–35 months	0.17 (0.09, 0.31)	< 0.001	0.09 (0.02, 0.36)	0.001
36–47 months	0.13 (0.07, 0.26)	< 0.001	0.04 (0.002, 0.60)	0.002

^**a**^Cumulative incidence ratios was adjusted for sex, study area using Poisson regression model with Generalized Estimating Equations (GEE)

## Discussion

A package of community-based interventions for drowning prevention was successfully implemented on a large-scale under real world conditions for over 100,000 children aged 9–47 months in rural Bangladesh. The package of interventions was associated with a significant reduction in the incidence of drowning deaths among children aged 12–47 months compared to baseline rates. The interpretation of findings from a pre-post comparison requires the assumption that the mortality reductions were not entirely due to a secular trend. With smaller family sizes and improvements in living standards such trends are not implausible. However, a review of very recent evidence shows that there are no signs that drowning mortality has been declining substantially in rural Bangladesh over the last 10 years (Rahman et al. 2019). Further, analysis of historical childhood mortality in parts of the study area showed that while all-cause mortality had declined remarkably for children aged 12–47 months over a 14 years period (between 1998 and 2012), drowning-specific mortality as a whole has remained largely unchanged (Alonge et al. 2017a). Hence, any observed effect in this study (beyond 2013) is unlikely due to trend.

In assessing which components of the intervention was most impactful, our study design faced the challenge that parents ultimately self-selected whether they would obtain a playpen or have their child actually attend a creche. By comparing groups of households by treatment category we sought to minimize the role of selection bias under the assumption that factors driving parental choices would remain constant. Subject to these caveats we found that creche was effective in reducing the risk of drowning for all children aged 12–47 months. The only previous study conducted to test the effectiveness of a crèche for drowning prevention had also concluded that the crèche was effective for reducing drowning deaths among children 1–5 years, with a similar effect size as found in our study (Rahman et al. 2012). However, the crèche package tested in that previous study combined other interventions such as playpens for younger children < 18 months, social autopsy, community education provided through injury committees and home hazard risk assessments, and was not able to demonstrate the effect of the various components of the intervention package targeted to children of different age groups (Rahman et al. 2012). In this study, we separated the creche package of interventions from the prior study into three streamlined intervention components: playpen-only, creche-only, and creche plus playpen, and combined each with community education provided by injury prevention committees. Further, we have attempted to determine the independent effect of these constituent components for drowning prevention among under-five children of different ages. We have also examined the effect of the creche component on a much larger scale (including over 100,000 children compared to about 18,500 children in the previous study). Hence, this current study moves the knowledge in the drowning literature closer to showing independent effectiveness of the crèche for drowning prevention.

Drowning among under-five children in Bangladesh is age-dependent, and the drowning risk within different age intervals among under-five children is not equivalent (Ahmed et al. 1999). Hence, it is important to identify the differential effect of specific interventions on children of different age groups. The crèche was effective for drowning prevention among children aged 12–23 months, 24–35 months and 36–47 months. The crèche protective effect may be conferred via four main mechanisms during the peak daily period for drowning (9 am – 1 pm) in rural Bangladesh: 1) provision of a safe environment from water hazard for at-risk children (Sleet and Gielen 2007), 2) provision of increased level of supervision (with respect to proximity, attentiveness and continuity of supervision) (Saluja et al. 2004), 3) institutionalization of drowning risk perceptions and 4) eliminating variability in prevention practices across at-risk population (Hubbard 2009), and provision of safety education in a developmentally appropriate way (Bandura 1989). The creche intervention systematically partitions childhood drowning threats by time, space and activities by keeping the children engaged in the creches between 9 am – 1 pm (the peak daily period for drowning), and targets supervision resources (including risk avoidance and reduction) for handling those threats at the same level across all at-risk populations. Further reviews of our data showed that all deaths recorded for children under the crèche program were outside of the 9 am – 1 pm period when the children were not under the protective environment of the crèche. By the same mechanisms, the crèche is also able to protect children from other types of injury fatalities while providing long-term societal benefits (such as those seen in education and labor) resulting from early childhood development and livelihood activities (Richter et al. 2016; Nair et al. 2017). These positive externalities are important for a socially-engaged drowning prevention program, unlike the case with other drowning interventions e.g. pool fencing. Thus, extending the cross-sector benefits of early childhood programs to injury prevention provides an additional impetus for investment in such programs across LMICs (Richter et al. 2016).

We could not establish the effectiveness (or lack thereof) of the playpen alone (or in combination with the creche) due to a limited sample size, but did observe higher drowning rates for those children under the playpen-only package (aged 9–24 months), similar to rates reported in the literature for children aged 0 -11 months in rural Bangladesh (Rahman et al. 2019). While the playpen intervention appears straightforward, it requires proactive use on the part of the caregiver for it to be effective. Caregivers were expected to use the playpen intervention as needed, and as frequently as they perceive any drowning risk for their children. They were provided instructions on how to use the playpen, but the instructions did not require them to use the playpen during the specific period of day when children were at most risk of drowning. The variability in drowning risk perceptions (and implementation of prevention practices, including playpen use) among caregivers may impact on the effectiveness of the playpen for drowning prevention. The creches on the other hand sequestered all children under adult supervision during the daily peak period for childhood drowning, irrespective of the variability in drowning risk perceptions and implementation of prevention practices among caregivers (as long as those caregivers had made the singular decision to bring the children to the creches). For example, further review showed that out of the 22 deaths that occurred for children under the playpen-only category, 12 (55%) occurred during the four-hour period of 9 am – 1 pm, almost exclusively among young children aged 12–18 months old. It appears that most caregivers may not have recognized the need to use the playpen during that period of day (9 am – 1 pm). In fact, only 5 of the 22 children that drowned were reported to be using the playpen during the last follow-up visit to the families, and the common reason reported for non-use was that “child does not want to stay inside playpen”.

Caregivers may have also assumed a false sense of security because of the presence of the playpen (and other project activities in their communities), and be willing to take more risk in their routine decisions around drowning hazards (risk compensation) (Hedlund 2000), inadvertently for children who are more prone to drowning as observed in other injury and non-injury prevention studies (Adams and Hillman 2001; Henderson et al. 2011; Hölmstrom 1979; Sagberg et al. 1997). Such behaviors may have contributed to the higher drowning incidence rates among children aged 0–11 months and those under the playpen-only category. Also, caregivers preferred the plastic playpen to the wooden playpen because of the former’s lighter weight, colorful appearance, and ease of mobility, and this could have led to differential usage and mortality rates comparing these two types of playpen. However, this study could not explore these differences due to limited sample size and power. Further studies would be needed to explore the effectiveness and optimal implementation arrangements for use of playpens alone for childhood drowning prevention.

It is important to highlight that while both creche and playpen involved community engagement through the activities of the UIPC and VIPC, the implementation of the crèche required more active involvement of the VIPC at various phases. Through the VIPC, the community participated actively in establishing the crèches, recruiting crèche mothers and children, and providing support to the day-to-day running of the crèches. Hence, the VIPC created a platform for effective delivery of crèche services through social action. Such social actions have been previously recognized as important factors for improving service delivery and health outcomes, and for far-reaching community-wide effect (Peters et al. 2009). Additional interviews conducted with caregivers suggested that they preferred the creche over the playpen given that the creche provided additional benefits, especially early childhood learning and opportunity for caregivers (mainly women) to earn supplemental income outside of the home.

We found that infant deaths were unexplainably higher during the implementation period compared to the baseline. It is possible that the baseline rates were underestimated given the potential for information and recall bias at baseline relative to data collected through active surveillance over a much longer implementation period. The preponderance of rivers in Area 2 may have also played a role in these infant deaths – since the deaths disproportionately happened in Area 2. We explored gender analysis and found that the proportionate deaths were not significantly different comparing male and female infants, and we do not have any reasonable evidence to suggest that the intentional deaths of female infants or other forms of infanticide took place during the implementation period.

This study has limitations. Caregivers self-selected into different treatment categories given that the study was not randomized; and a differential mortality rate may exist between children whose parents chose different intervention packages due to unobserved factors that may have been associated with higher or lower drowning risk. Hence, interpreting patterns of drowning mortality between treatment categories may be biased. Two types of self-selection bias might create two types of bias. Under adverse selection, parents at most risk due to social vulnerability and reduced risk perception would be less likely to adopt creches or playpens, and this would associate the intervention packages with a higher drowning hazard. On the contrary, under positive selection, vigilant parents who had already done everything in their power to lower their child’s drowning risk would selectively adopt the creches and playpens as well, and this would associate the intervention packages with spuriously lower drowning hazard. Ex ante, we cannot predict which form of selection bias would occur or which would dominate. We can only advise extreme caution in interpreting the patterns in drowning mortality when categorized into the treatment categories that parents ultimately selected. One strategy that we used to help to reduce selection bias was be to compare the pre-intervention drowning rates for each of the treatment categories ascertained by the 1 year retrospective survey at baseline to the post-intervention drowning rates that occurred during the intervention for the same household by each category. Under the assumption that the unobservable reasons that parents chose the way they did remained constant over time, this pre-post comparison would reduce the impact of selection bias in this study.

Misclassification of drowning deaths under the different treatment categories and age group is possible given the variable data quality upon which the analysis was based. To minimize misclassification errors, information on the dates of birth and death were triangulated from multiple data sources for all study participants, and manually cross-checked for all incident cases. In addition, all information for incident cases under the playpen category was cross-validated by re-interviewing respondents at the time of data analysis.

There were also differences in the implementation of the interventions due to different implementing agencies, and in the type of playpens used in Area 1 and 2. The study findings also suggest that the interventions may have had variable level of intensity across the study population, which is not uncommon for large-scale programs (Kilbourne et al. 2007). For instance, drowning risk was significantly higher in Area 2 compared to Area 1, which may be explained by the greater proximity of sub-districts in Area 2 to rivers. The magnitude of decline in the drowning risk was also higher in Area 2, though the rate of declining mortality over the implementation period was similar for both areas (as shown in Table 1). Such variations in implementation intensity and other factors may reflect the dynamics of implementing the interventions under real-world conditions and provide for a more robust conclusion about the effect of the intervention at scale (Kilbourne et al. 2007). Thus, we had adjusted for Area 1 or 2 in estimating the effect of the interventions as highlighted in Tables 2 and 3.

The findings of this study can be reasonably generalized to the non-studied unions and other rural sub-districts in Bangladesh given similarities in their contexts with the study area. One factor that might not be generally available in all parts of Bangladesh would be the presence of an implementing NGO with the capacity to convene stakeholders and engage the community members themselves in promoting the educational messages and utilization of the creches. The study findings could also be generalized to other LMICs with similar characteristics and implementation capacity to our study areas in rural Bangladesh.

## Conclusions

Community-led crèche interventions are effective at scale for reducing drowning deaths among children in a rural LMIC setting like Bangladesh and should be considered for further scale up to reduce the global burden of drowning among under-five children especially in LMICs. Playpen-only may not be effective for drowning prevention among children, and additional implementation studies are required to further explore playpen implementation and other barrier strategies for childhood drowning prevention in LMICs. More research studies are also needed to explore the dose-response effect of the creche package for different age groups and to further streamline the package to identify its simplest form. We recognize that the equivalence of the level of supervision provided by paid creche workers may not be available or affordable to most caregivers in rural Bangladesh. Therefore, it is important for government, donors and civil society organizations to recognize creches as important social services which can help save lives from drowning, and not leave the provision of these services to the market entirely, but play their part in providing these services at scale.

## Supplementary information

**Additional file 1: Appendix 1.** Instructions to caregivers on the use of the playpens.

## Data Availability

The datasets used/or analysed during the current study are available from the corresponding author on reasonable request.

## Abbreviations

LMICs: Low- and middle-income countries
NGO: Non-governmental organizations
SoLiD: Saving of Lives from childhood Drowning
UIPC: Union Injury Prevention Committees
USD: United States Dollars
VIPC: Village Injury Prevention Committees

## References

[CR1] Adams J, Hillman M (2001). The risk compensation theory and bicycle helmets. Inj Prev.

[CR2] Ahmed MK, Rahman M, van Ginneken J (1999). Epidemiology of child deaths due to drowning in Matlab, Bangladesh. Int J Epidemiol.

[CR3] Alonge O, Agrawal P, Talab A, Rahman QS, Rahman AF, El Arifeen S, Hyder AA (2017). Fatal and non-fatal injury outcomes: results from a purposively sampled census of seven rural subdistricts in Bangladesh. Lancet Glob Health.

[CR4] Alonge O, He S, Hoque DE, Salam SS, Islam I, El-Arifeen S, Hyder AA (2017). Shifting disease burden in low and middle-income countries: a 14-year survival analysis of childhood mortality in Bangladesh. J Epidemiol Community Health.

[CR5] Bandura A, Vasta R (1989). Social cognitive theory. Annals of Child Development. Vol. 6. Six theories of child development.

[CR6] Chowdhury AM, Bhuiya A, Chowdhury ME, Rasheed S, Hussain Z, Chen LC (2013). The Bangladesh paradox: exceptional health achievement despite economic poverty. Lancet.

[CR7] Gain P, Moral S, Raj P, Sircar L (2002). Bangladesh environment: facing the 21^st^ century.

[CR8] Hedlund J (2000). Risky business: safety regulations, risk compensation, and individual behavior. Inj Prev.

[CR9] Henderson L, Clements A, Damery S, Wilkinson C, Austoker J, Wilson S (2011). ‘A false sense of security’? Understanding the role of the HPV vaccine on future cervical screening behaviour: a qualitative study of UK parents and girls of vaccination age. J Med Screen.

[CR10] Hölmstrom B (1979). Moral hazard and observability. Bell J Econ.

[CR11] Hubbard D. The failure of risk management: why It's broken and how to fix it: Wiley; 2009.

[CR12] Hyder AA, Alonge O, He S (2014). Saving of children's lives from drowning project in Bangladesh. Am J Prev Med.

[CR13] Hyder AA, Alonge O, He S (2014). A framework for addressing implementation gap in global drowning prevention interventions: experiences from Banglades. J Health Popul Nutr.

[CR14] Hyder AA, Borse NN, Blum L, Khan R, El Arifeen S, Baqui AH (2008). Childhood drowning in low- and middle-income countries: urgent need for intervention trials. J Paediatr Child Health.

[CR15] International Centre for Diarrheal Disease Research, Bangladesh (icddr, b) (2007). Health and Demographic Surveillance System – Matlab. Registration of Health and Demographic Events 1992–2013.

[CR16] Kilbourne AM, Neumann MS, Pincus HA, Bauer MS, Stall R (2007). Implementing evidence-based interventions in health care: application of the replicating effective programs framework. Implement Sci.

[CR17] Liu L, Johnson HL, Cousens S, Perin J, Scott S, Lawn JE, Rudan I, Campbell H, Cibulskis R, Li M, Mathers C (2012). Global, regional, and national causes of child mortality: an updated systematic analysis for 2010 with time trends since 2000. Lancet.

[CR18] Nair D, Alonge O, Derakhshani Hamadani J, Sharmin Salam S, Islam I, Hyder AA (2017). Developmental assessments during injury research: is enrollment of very young children in crèches associated with better scores?. Int J Environ Res Public Health.

[CR19] National Institute of Population Research and Training (NIPORT) (2016). Bangladesh Demographic and Health Survey 2014.

[CR20] Peden M, Oyegbite K, Ozanne-Smith J, Hyder AA, Branche C, Rahman F, Bartolomeos K (2008). World report on child injury prevention.

[CR21] Peters DH, El-Saharty S, Siadat B, Janovsky K, Marko V (2009). Improving health service delivery in developing countries: from evidence to action.

[CR22] Petrass LA, Blitvich JD, Finch CF (2011). Lack of caregiver supervision: a contributing factor in Australian unintentional child drowning deaths, 2000-2009. Med J Aust.

[CR23] Rahman A, Jagnoor J, ul Baset K, Ryan D, Ahmed T, Rogers K, Hossain MJ, Ivers R, Rahman AF (2019). Vulnerability to fatal drowning among the population in Southern Bangladesh: findings from a cross-sectional household survey. BMJ Open.

[CR24] Rahman A, Rahman AF, Shafinaz S, Linnan M (2005). Bangladesh Health and Injury Survey Report on Children.

[CR25] Rahman F, Bose S, Linnan M (2012). Cost-effectiveness of an injury and drowning prevention program in Bangladesh. Pediatrics..

[CR26] Richter LM, Daelmans B, Lombardi J, Heymann J, Boo FL, Behrman JR, Lu C, Lucas JE, Perez-Escamilla R, Dua T, Bhutta ZA (2016). Investing in the foundation of sustainable development: pathways to scale up for early childhood development. Lancet.

[CR27] Sagberg F, Fosser S, Sætermo IA (1997). An investigation of behavioral adaptation to airbags and antilock brakes among taxi drivers. Accid Anal Prev.

[CR28] Saluja G, Brenner R, Morrongiello BA, Haynie D, Rivera M, Cheng TL (2004). The role of supervision in child injury risk: definition, conceptual and measurement issues. Inj Control Saf Promot.

[CR29] Sleet D, Gielen A. Behavioral Intervention for Injury and Violence Prevention. In: Doll L, Bonzo S, Sleet D, Mercy J, editors. Handbook of Injury and Violence Prevention: Springer Science & Business Media; 2007. p. 405–7.

[CR30] StataCorp (2015). Stata statistical software: release 14.

[CR31] United States CPSC. Standards for Play-Yards Available at https://www.cpsc.gov/Regulations-Laws%2D%2DStandards/Rulemaking/Final-and-Proposed-Rules/Play-Yards/. n.d.

[CR32] van Beeck EF, Branche CM, Szpilman D, Modell JH, Bierens JJ (2005). A new definition of drowning: towards documentation and prevention of a global public health problem. Bull World Health Organ.

[CR33] World Health Organization (2014). Global report on drowning: preventing a leading killer.

[CR34] World Health Organization (2016). Global Health Estimates 2015.

[CR35] Ya F, Long D, Jaung MS, Xiaoxuan C, Songlin Y, Huiyun X (2007). Child drowning deaths in Xiamen city and suburbs, People's Republic of China, 2001-5. Injury Prevention.

