# Supplementary material for: Large-scale evaluation of interventions designed to reduce childhood Drownings in rural Bangladesh: a before and after cohort study
Source: Inj Epidemiol. 2020 May 11;7:17. doi: 10.1186/s40621-020-00245-2 (PMC7212604; doi:10.1186/s40621-020-00245-2)
Supplement: Supplementary file 1 — Additional file 1: Appendix 1. Instructions to caregivers on the use of the playpens. [file 40621_2020_245_MOESM1_ESM.docx]

**Appendix 1:** Safety guideline for the use of playpen

| **Safety guideline for the use of playpen**   - Place the playpen in a safe location away from fire, heat sources or another hazardous environment - Keep the playpen away from cords, clothes line, and hanging ropes/drapes as these can pose strangulation hazard - Place the playpen at ground level and on an even surface always - Do not use pillows, blankets, wraps, quilts or sheets when the child is placed in the playpen as these can cause suffocation - If a mattress is used in the playpen make sure that it is firm and tight fitting and that there is no more than two fingers space between the mattress and the side of the playpen - Do not place large toys, boxes or any large objects in the playpen as these can be used by the child to climb out of the playpen - Toys and other items should not be tied at the corner or on the top of the playpen railing as these can be a strangulation hazard - Check the railing for holes and tears as children may bite the top railing during teething; discontinue the use of the playpen if these holes cannot be repaired - If a sleeping child is placed in the playpen, put the child on its back and do not use bedding, cushion, pillow, blankets, wraps, sheets or soft toys as these may cause suffocation - Do not give children plastic wrappings or plastic bags to play with when in playpen or otherwise - Do not tie anything around child’s neck (e.g. threads, necklace, bibs, pacifiers) because if such an item catches somewhere in the playpen it can lead to strangulation - Do not cover the playpen when it is in use, i.e. when a child is kept in it - Do not use the playpen if it is unstable - Do not use the playpen if there are problems with its structural integrity - If the child can climb out of the playpen, do not use it as it can cause fall injuries to the child - Do not use the playpen if you notice any splits, cracks or other defects - Do not use the playpen if its surface is rough; has sharp edges, corners or uneven planes, as these can cause injuries to the child - Do not use the playpen if it has a missing slat to avoid head entrapment and/or strangulation - Place only one child in the playpen at a time - Keep the child in the playpen for short periods of time - Playpens assist in parental supervision efforts and are not meant to replace it. Hence, check the child frequently whenever s/he is placed in the playpen |
| --- |
